# Network reorganization and breakdown of an ant–plant protection mutualism with elevation

**DOI:** 10.1098/rspb.2016.2564

**Published:** 2017-03-15

**Authors:** Nichola S. Plowman, Amelia S. C. Hood, Jimmy Moses, Conor Redmond, Vojtech Novotny, Petr Klimes, Tom M. Fayle

**Affiliations:** 1 Faculty of Science, University of South Bohemia, Ceske Budejovice, Czech Republic; 2 Institute of Entomology, Biology Centre of Czech Academy of Sciences, Ceske Budejovice, Czech Republic; 3 New Guinea Binatang Research Center, Madang, Papua New Guinea; 4 Department of Zoology, University of Cambridge, Cambridge, UK; 5 University of Papua New Guinea, Port Moresby, Papua New Guinea

**Keywords:** altitudinal gradients, biotic defence, global change, herbivory, myrmecophyte, network specialization

## Abstract

Both the abiotic environment and the composition of animal and plant communities change with elevation. For mutualistic species, these changes are expected to result in altered partner availability, and shifts in context-dependent benefits for partners. To test these predictions, we assessed the network structure of terrestrial ant-plant mutualists and how the benefits to plants of ant inhabitation changed with elevation in tropical forest in Papua New Guinea. At higher elevations, ant-plants were rarer, species richness of both ants and plants decreased, and the average ant or plant species interacted with fewer partners. However, networks became increasingly connected and less specialized, more than could be accounted for by reductions in ant-plant abundance. On the most common ant-plant, ants recruited less and spent less time attacking a surrogate herbivore at higher elevations, and herbivory damage increased. These changes were driven by turnover of ant species rather than by within-species shifts in protective behaviour. We speculate that reduced partner availability at higher elevations results in less specialized networks, while lower temperatures mean that even for ant-inhabited plants, benefits are reduced. Under increased abiotic stress, mutualistic networks can break down, owing to a combination of lower population sizes, and a reduction in context-dependent mutualistic benefits.

## 1. Introduction

The structure and composition of plant and animal communities are affected by both the biotic and the abiotic environment [1]. Every species is involved in a myriad of beneficial, antagonistic and neutral interactions with multiple other species, and the strength and direction of these interactions is often dependent on the environmental context [1]. Beneficial interactions are widespread, abundant and important in the structuring of communities [2] to the extent that they can determine the geographical ranges of species [3] owing to the context-dependent costs and benefits for the species involved [4]. Climatic context may be particularly important in determining the strength of these mutualistic interactions [5]. Shifts in mutualistic interaction networks in relation to latitude are well known, with interactions being less specialized in the tropics owing to high diversity of plant partner species, which in turn may be related to climate [6]. Examining the distribution of mutualistic species over natural temperature gradients on mountains is the next step towards understanding how climate can shape these networks, and potentially allows comparisons in responses between latitudinal and altitudinal gradients [7,8].

Among the best-studied mutualistic networks are ant–plant mutualisms [9], the outcomes of which can be highly context-dependent [10]. Hence, these interactions are particularly interesting to study in relation to shifts in the abiotic environment. Ant-plants, or myrmecophytes, provide ants with nesting space or food rewards such as extra-floral nectaries (EFNs) and protein-rich food bodies, in return for protection against herbivores or trimming of encroaching vegetation [9]. In some cases, symbiotic ants can also provide nitrogen for plants through absorption of ant waste [11,12]. In the tropics, where ants are most diverse and numerous, these mutualisms become more common, with greater incidence of both EFN-bearing plants [13] and those with structures to house ant colonies (domatia) [14]. The costs and benefits to plants of hosting ants or providing food rewards can depend on biotic factors such as herbivory pressure, or the identity of the colonizing ant species, which can vary in their effectiveness of protection [15], and on abiotic factors such as light or nutrient limitation [16,17]. As such, the strength of the mutualism is expected to depend on the selective pressures facing the plant, which are affected by the environment. If costs outweigh benefits for at least one partner, then this can result in the breakdown of the mutualism, with one partner becoming parasitic, or with the interaction being abandoned [18].

Although network structure in ant–plant mutualisms has been reasonably well documented, much less is known about shifts in these interactions with elevation, and how this might affect partner benefits (but see [19–21]). Partner availability may play an important role in such shifts. With elevation, ants decrease in abundance and may be less important as predators of herbivores [22]. In addition to decreased ant-partner availability, there can be changes in the effectiveness of persisting ant partners. For example, ants protecting Neotropical EFN-bearing *Inga* species are less active and less effective in the uplands, resulting in greater herbivore damage [19]. By contrast, *Piper immutatum,* a Neotropical domatia-bearing plant, experiences similar levels of herbivory throughout its elevational range [21]. As previous work has focused on single ant or plant species [19,20], it is not known how whole networks change with elevation, and the associated effects on plant benefits. If abundance and species richness of plant–ants and ant–plants declines with temperature at higher elevations, the structure of mutualistic networks will also change. At a network level, decreased specialization could occur as a result of reductions in population sizes of some partner species, and complete loss of others, reducing possibilities for partner choice. This would result in a greater degree of connectance (a greater proportion of possible links between species are realized) [23], and a corresponding lower modularity (the degree to which the network is divided into discrete groups of interacting species) [24]. These effects are distinct from changes in network structure that occur only as a result of changes in network size, and also from spatial turnover of networks (independent of any environmental factors), in which only a central core of generalist species persist where ant-plants are surveyed at a single elevation [25]. Furthermore, associations with the ‘wrong’ partner species might reduce the effectiveness of plant protection, resulting in increased herbivory damage [15]. A similar effect is also expected if there are reductions in patrolling rates within-ant species as temperatures decrease.

We studied a community of terrestrial (non-epiphytic) ant-plants and their ant inhabitants in primary forest from 700–1600 m.a.s.l. in Papua New Guinea (PNG) to investigate: metres above sea level (m.a.s.l.) (i) how ant–plant interaction networks change with elevation, (ii) how ant protective behaviour on a focal species, *Myristica subalulata*, changes with elevation, and (iii) whether there are correlated changes in plant herbivory damage.

## 2. Methods

### (a) Study site

We censused a community of terrestrial understorey ant-plants in June–August 2013 in wet primary rainforest on the slopes of Mount. Wilhelm near Numba village in Madang Province, PNG (5° 43′ 18″ S, 145° 16′ 12″ E; electronic supplementary material, figure S1). The area experiences a mild dry season between late June and early August. Temperature drops linearly with elevation from a daily mean of 27.4°C at 200 m.a.s.l. at approximately 0.58°C 100 m^−1^ (electronic supplementary material, figure S2).

### (b) How do ant–plant interaction networks change with elevation?

**Figure 1.**
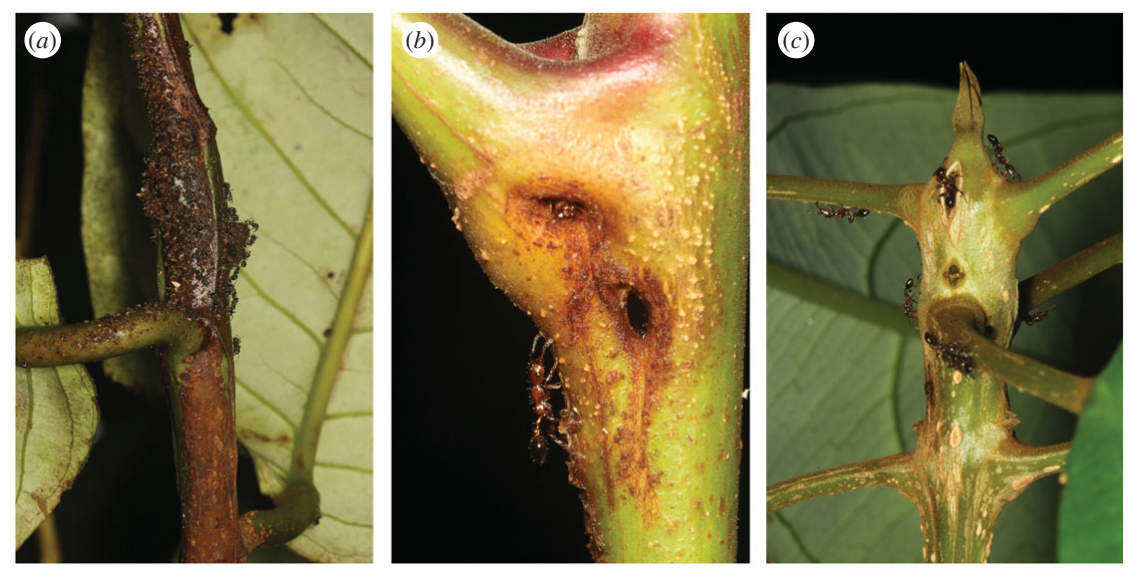
Domatia of the three most abundant ant-plant species in our study. (*a*) Swollen stem domatia of *Myristica subalulata* being excavated by *Anonychomyrma* ants, (*b*) entrance holes of a *Chisocheton lasiocarpus* domatium occupied by *Podomyrma* sp. 3, and (*c*) *Podomyrma* sp. 3 patrolling the swollen stem domatia of *Ryparosa amplifolia*.

We established ten 0.15 ha transects (150 × 10 m), at elevational intervals of 100 m, from 700 to 1600 m.a.s.l., the highest point of the local topography. This spans the rapid decline in ant species richness observed on many tropical mountains [26,27], including Mount Wilhelm [28]. We did not sample forests below 700 m.a.s.l., which were subject to human disturbance. In each transect, we examined all understorey trees (up to 15 m height) for entrance holes and ant activity in stems, branches or other pre-formed domatia and tagged all ant-inhabited trees (*n* = 386; figure 1).

Additionally, we censused all unoccupied individuals more than or equal to 1 m in height (*n* = 102) of the three most commonly inhabited species *Ryparosa amplifolia* (Achariaceae), *Myristica subalulata* (Myristicaceae) and *Chisocheton lasiocarpus* (Meliaceae). We identified each tree to species, recording height and diameter at breast height (DBH). Plant vouchers are deposited at New Guinea Binatang Research Center, Madang, PNG. We conducted transects every 100 m in elevation, rather than at fewer elevations with more replications, and since we tested elevational trends, local irregularities owing to unreplicated transects should manifest as outliers. The unimodal abundance of most ant-plants across elevations (see Results) indicates that we captured shifts in distributions with elevation reasonably well. However, because our results relate to only a single mountain, we are cautious in their interpretation.

Where possible without causing damage to the plant (and hence compromising plant-benefit assays; see below), we collected 1–15 ants in absolute ethanol from each inhabited tree (355 of 386 individuals). Where ants were resident but not collected the species was assigned as ‘uncertain’. Ants were identified to morphospecies and species where possible, with species delineations refined using existing reference collections and DNA barcoding (electronic supplementary material, appendix S3).

All statistical analyses were performed in R [29]. We generated bipartite networks for each elevation and calculated the metrics *Connectance* (realized proportion of possible links), *generality* (plant species per ant species), *vulnerability* (ant species per plant species), *modularity* (see [30]) and *network specialization* (H_2_′; deviation from random partner choice [31]) with the function ‘networklevel’ in the R package bipartite [32]. Observed H_2_′ was compared with randomly expected values (Monte Carlo statistics; electronic supplementary material, table S3). Connectance and generality metrics, respectively, were square root and log transformed to meet normality assumptions before testing their relationship with elevation (linear or quadratic regressions depending on fit as measured using Akaike information criterion (AIC)). Vulnerability, H_2_′ and modularity residuals were not improved by transformation so their relationship with elevation was tested using the non-parametric Hoeffding's D statistic [33]. Variation in abundance can account for changes in network metrics [34]. To account for the effects of decreasing ant-plant abundance with elevation, we calculated 95% prediction intervals for all metrics based on repeated rarefaction of a pooled low-elevation community (700 and 800 m combined) to match abundances at higher elevations (1000 replications per elevation).

### (c) How do ant patrolling, herbivore detection, recruitment and attack change with elevation?

To understand how ant protective behaviour changes with elevation, we focused on the interaction between the most common myrmecophyte, *M. subalulata*, and the most common genus of resident ants, *Anonychomyrma*, both spanning the entire elevational range. *Myristica subalulata* is a widespread understorey tree in New Guinea [35]. Although lacking EFNs and food bodies, when occupied by ants *M. subalulata* frequently has honeydew producing coccids inside its pre-formed domatia [35,36].

**Figure 2.**
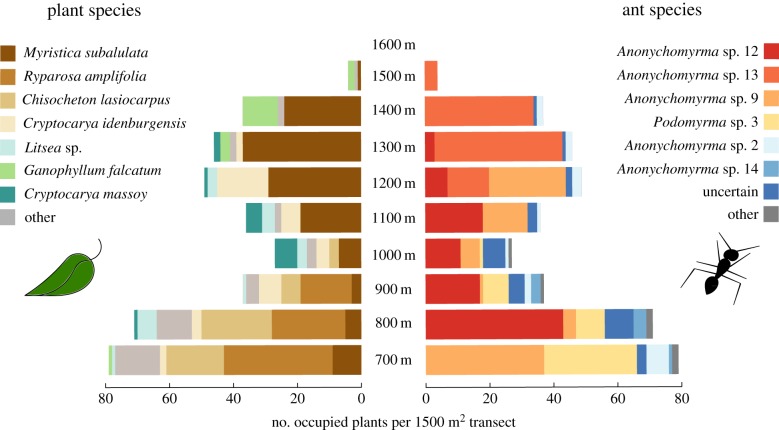
Distribution of ant-inhabited plant species (*n* = 386 trees) and their ant occupants from 700 to 1600 m.a.s.l. No ant-inhabited trees were found at 1600 m. Bars on the left indicate the number of ant-occupied individuals per tree species. Bars on the right indicate the number of occupied trees per ant species. Individual plants were only ever occupied by one species of ant, but most plant species were inhabited by multiple species of ant across multiple plant individuals. Where ants were observed in domatia, but could not be collected, they were recorded as ‘uncertain’.

Ant behaviour was assessed from 700 to 1400 m.a.s.l. (*n* = 80), because ant occupancy was rare above 1400 m (figure 2; electronic supplementary material, figure S3). We surveyed 10 trees per elevation between 10.30 and 15.00, when ants were most active. Trees 0.4–9.0 m in height were selected at random within transects, supplemented from the surrounding area when necessary. To assess active leaf patrolling by resident ants, we randomly selected two mature and two young leaves per tree to control for leaf age (not all trees had both; *n* = 74 and 28 trees respectively), and instantaneously recorded the number of ants. Young leaves were defined as smaller, paler and fleshier than mature leaves, and were selected only when fully expanded. The relationship of elevation and ant species with active leaf patrolling was tested with repeated measures ANOVA, using leaf age as a within-subject variable.

Following previous work [37], we assessed ant responses to simulated herbivory. A single live worker termite, *Microcerotermes* sp. (not a natural herbivore of *M. subalulata*, but uniform in size, smell and lack of defences), hereafter referred to as the surrogate ‘herbivore’, and a paper control (0.5 × 0.5 cm) were pinned on 10 plants per elevation. The position was standardized to the second pair of leaves from the base of a randomly selected branch more than 1.5 m from ground level, 5 cm from the petiole along the midrib of different leaves. As only a minority of plants had young leaves (see above), at this position most leaves were mature and thus reasonably uniform in size. The control and treatment were alternated between left and right for every trial. We observed for 10 min to record:

(i) time until first discovery (ant touching paper/herbivore with antennae or mandibles);
(ii) time until arrival of first recruit (the second ant to locate the paper/herbivore);
(iii) time spent by any ants actively attacking the paper/herbivore; and
(iv) maximum number of ants on the leaf simultaneously.

Each metric was modelled as a function of elevation, tree height and ant species using repeated measures ANOVA to account for control and herbivore treatments on each tree. As the explanatory variables ant species and elevation are co-dependent, we present models for each predictor individually, and with all predictors present (electronic supplementary material, tables S4–14). Additionally, we individually modelled the two ant species that were most widespread across elevations to test separately for within-ant species effects (see the electronic supplementary material, S16–17).

### (d) Are there changes in herbivory damage with elevation that might be driven by changes in ant protection?

Herbivory was estimated visually for all trees less than or equal to 5 m in height by assigning each leaf to a damage category (0%, less than 5%, 5–33%, more than 33% missing leaf area). On trees with less than or equal to 50 leaves, estimates were based on all leaves, and on trees with more than 50 leaves, approximately every third leaf. For plotting herbivory and for testing repeatability (but not for the main analysis, see below), we estimated mean percentage herbivory per tree by using an abundance-weighted average of the midpoint of each herbivory category. N.S.P. performed estimates for the census data, and C.R. for the behavioural assay data, with 45 trees in common. Estimates were highly correlated between observers (Pearson's product–moment correlation; *t*_43_ = 5.11, *p* < 0.001). Though this method only provides a ‘snapshot’ measure of herbivory, and could not capture leaves that were completely missing, it reflects the damage accrued to leaves over their lifetime, and is appropriate for comparison over a landscape scale.

For the most common species (more than 10 occurrences: seven plant species, five ant species), herbivory was modelled as a function of elevation, tree height, ant species and tree species using ordinal logistical regression (clmm function, package ‘ordinal’), with leaf as a random factor and data as counts in ordered categories. Again, because ant species, tree species and elevation are co-dependent, we present models for each predictor individually, and with all predictors present (electronic supplementary material, tables S18–S26). Models were selected using AIC.

## 3. Results

### (a) How do ant–plant interaction networks change with elevation?

**Figure 3.**
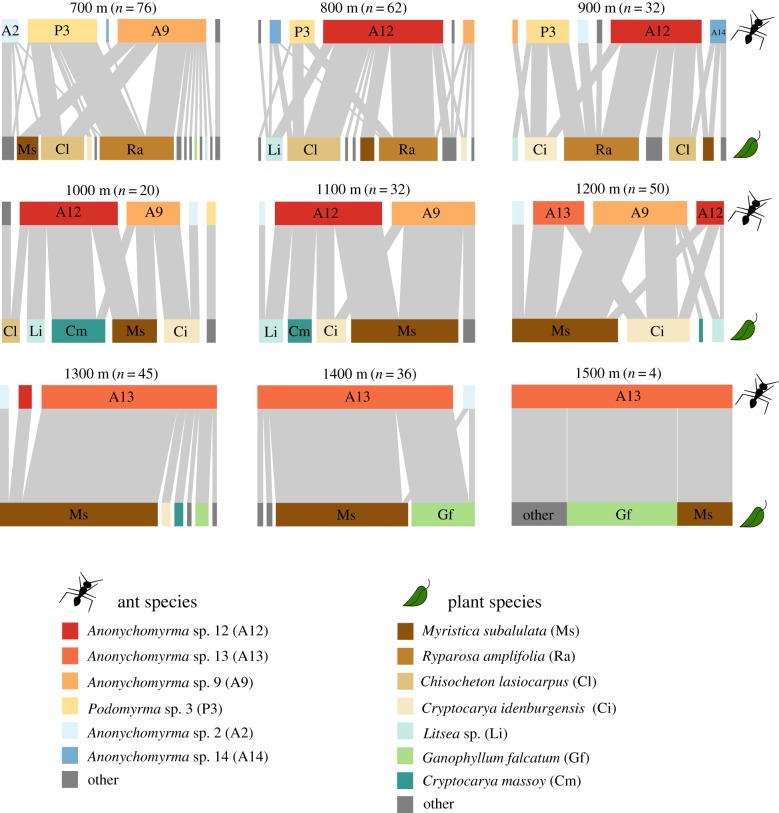
Bipartite interaction networks of ant-inhabited plants and their ant occupants from 700 to 1500 m.a.s.l. Upper blocks represent ant species, lower blocks represent plant species and connecting light grey bars indicate species interactions. Species with fewer than 10 occurrences are assigned as ‘other’ (grey blocks), but are not combined (i.e. the foodweb is fully resolved). Width of bars represents the proportion of the total community of ants or plants interacting at a given elevation. Note that the total abundance of ant-plants varied between elevations (sample size in brackets).

We found 23 species of ant-inhabited plants belonging to six families, and 10 species of ant inhabitants in five genera (figures 2 and 3; electronic supplementary material, tables S1 and S2). Ant inhabitation ranged from 700 to 1500 m.a.s.l., with no evidence of inhabited plants at 1600 m. Each individual plant was occupied by only one species of ant, presumably representing a single colony (no within-plant aggression was observed). Occupancy of the three most abundant plant species was high; 72%, 60% and 68% for *M. subalulata, C. lasiocarpus* and *R. amplifolia* respectively, though this varied with elevation, with some evidence for reduced partner availability at higher elevations (electronic supplementary material, figure S3*a*–*c*). Five species of *Anonychomyrma* (Dolichoderinae) and one species of *Podomyrma* (Myrmicinae) were the most common plant inhabitants. Ants from the genera *Colobopsis, Pheidole* and *Tetramorium* were also found inhabiting plants, but only rarely (less than three occurrences per ant species; electronic supplementary material, table S2).

**Figure 4.**
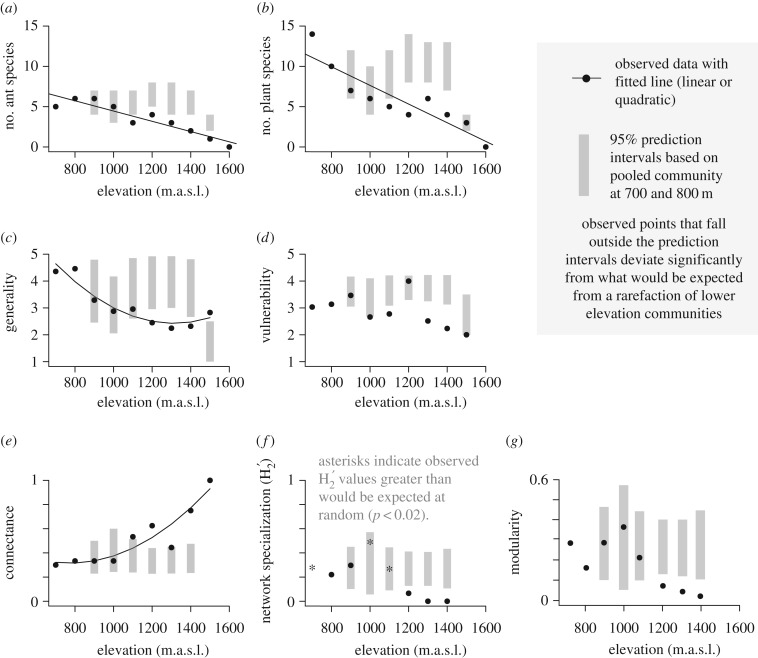
(*a*) Species richness of plant-inhabiting ants, (*b*) species richness of ant-inhabited plants, (*c*) generality, (*d*) vulnerability, (*e*) connectance, (*f*) network specialization (H_2_′) and (*g*) modularity of networks from 700 to 1600 m.a.s.l. Grey bars indicate the 95% prediction intervals based on rarefying a pooled community from 700 and 800 m, and black circles indicate observed data fitted with linear (*a*,*b*) and quadratic regressions (*c*,*e*). In panels (*d*), (*f*) and (*g*) non-parametric analyses were used, hence no line of best fit is presented, although significant relationships with elevation were detected. Asterisks in panel (*f*) indicate observed H_2_′ values which differed significantly from what would be expected if ant species colonized plant species at random within elevations.

With increasing elevation, the species richness of both plants and their ant inhabitants decreased (linear regressions; plants: *p* < 0.001, *r*^2^ = 0.80, *F* = 36.3; ants: *p* < 0.001, *r*^2^ = 0.85, *F* = 52.5; figure 4*a*,*b*). Although generality (quadratic regression; *p* = 0.001, *r*^2^ = 0.85, d.f. = 6; figure 4*c*) and vulnerability (Hoeffding's *D*; *p* = 0.006, *D* = 0.23, *n* = 9; figure 4*d*) decreased with elevation, there was an increase in connectance with elevation (quadratic regression; *p* = 0.003, *r*^2^ = 0.81, d.f. = 6; figure 4*e*). Network specialization (H_2_′) (Hoeffding's *D*; *p* *=* 0.031, *D* = 0.15, *n* = 8; figure 4*f*) and modularity (Hoeffding's *D*; *p* = 0.002, *D* = 0.34, *n* = 8; figure 4*g*) both declined with elevation. The decrease in modularity at higher elevations corresponds to an increase in the dominance of *Anonychomyrma* sp. 13, which interacted with all plant species present at those elevations. At 700 m, 1000 m and 1100 m, network specialization (H_2_′) was greater than would be expected at random (*p* < 0.02). All network metrics changed more than would be expected from rarefaction of lowland ant-plant communities (grey bars in figure 4*a*–*g*) although results were less consistent at 1500 m owing to small sample size. When 1500 m data were excluded, the effect of elevation on generality and connectance remained significant (*p* = 0.001 and 0.008 respectively), but not on vulnerability (*p* = 0.136). Networks lacked discrete compartments, i.e. there were no groups of species that were entirely disconnected with the rest of the network (figure 3), and the overall network specialization was low compared with other myrmecophytic (i.e. domatia-bearing) networks (H_2_′ ≤ 0.5) [23].

### (b) How do ant patrolling, herbivore detection, recruitment and attack change with elevation?

More ants patrolled young *M. subalulata* leaves than mature leaves (repeated measures ANOVA; *F*_24_ = 15.1, *p* < 0.001; electronic supplementary material, figure S4). There was no effect of elevation on patrolling of mature leaves (ANOVA; *F*_74_ = 0.18, *p* = 0.673) or young leaves (ANOVA; *F*_28_ = 1.03, *p* = 0.32) but patrolling numbers differed between ant species (*p* < 0.001, *F*_74_ = 5.70).

**Figure 5.**
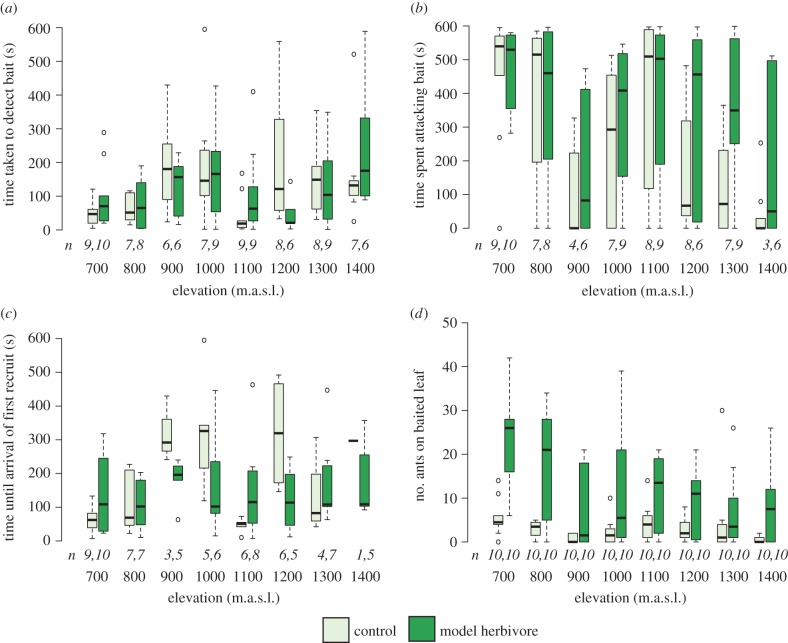
Responses of *Anonychomyrma* ants inhabiting *Myristica subalulata* trees to a surrogate herbivore and control treatment from 700 to 1400 m.a.s.l. (*a*) Time taken to detect, (*b*) time spent attacking, (*c*) time until the first recruit contacted the control/herbivore, and (*d*) maximum number of ants observed on experimental leaf at one time. Sample size in italics. (Online version in colour.)

On *M. subalulata* 76% of controls and 79% of herbivores were detected by ant inhabitants (all were species in the genus *Anonychomyrma; n* = 140; electronic supplementary material, figure S5). For the three most abundant ant species (sp. 9, sp. 12 and sp. 13, inhabiting *n* = 20, 34, and 21 trees, respectively), there was no effect of elevation (repeated measures ANOVA; *p* = 0.078, *F*_54_ = 1.96) or of treatment (*p* = 0.758, *F*_54_ = 0.10) on detection time (figure 5*a*). However, detection time differed between ant species (*p* = 0.028, *F*_58_ = 3.81; electronic supplementary material, figure S6*a*).

Of those detected, all herbivores and 89% of controls were attacked. Time spent attacking declined with elevation (figure 5*b*, *p* < 0.001, *F*_66_ = 4.75), although attack times were variable at middle elevations. Ants spent more time attacking the herbivore than the control (*p* < 0.001, *F*_74_ = 18.38; figure 5*b*). Time spent attacking varied between ant species (*p* < 0.001, *F*_71_ = 11.09), with *Anonychomyrma* sp. 9 spending longest attacking both herbivores and controls (electronic supplementary material, figure S6*b*).

Of those that were attacked, further workers were recruited to 84% of herbivores and 77% to controls. Recruitment time differed among elevations (*p* = 0.021, *F*_42_ = 2.71) but did not differ between species or treatments (figure 5*c*; electronic supplementary material, figure S4*c*). The maximum abundance of ants simultaneously present on the leaf declined with elevation (*p* < 0.001, *F*_66_ = 3.09), with more ants on leaves in the herbivore treatment than the controls (*p* < 0.001, *F*_74_ = 69.0) (figure 5*d*). Maximum abundance also differed between ant species (*p* < 0.001, *F*_71_ = 9.60) with *Anonychomyrma* sp. 9 being the most abundant, (electronic supplementary material, figure S6*d*). When testing *Anonychomyrma* sp. 9 and 12, which were present at five or more elevations, we found no evidence for any within-species changes in any of the measured protective behaviours with elevation (electronic supplementary material, tables S16–S17).

### (c) Are there changes in herbivory damage with elevation that might be driven by changes in ant protection?

**Figure 6.**
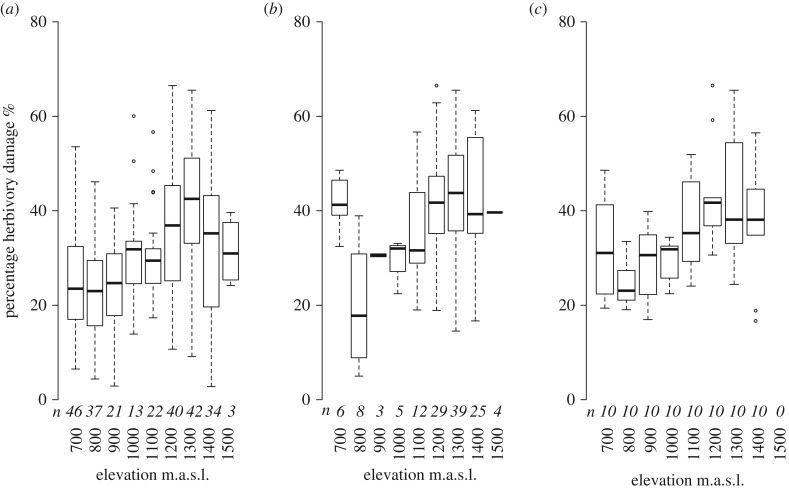
Percentage herbivory damage of (*a*) all ant-inhabited plants, (*b*) all inhabited individuals of *M. subalulata* in the transects, and (*c*) all individuals of *M. subalulata* at the time of baiting experiment from 700 to 1400 m.a.s.l. Leaves were assessed on all trees more than or equal to 5 m and categorized from 1 to 4 (0%, less than 5%, 5–33%, more than 33% damage, respectively). Counts across categories were converted into means for the purposes of plotting, using the midpoint for each category. Sample size in italics.

For the entire plant community, herbivory increased with elevation (ordinal logistic regression; *p* < 0.001, *z* = 54.6, *n* = 7584 leaves; 507 trees; figure 6), and differed between ant species (*p* < 0.001; electronic supplementary material, table S18) and tree species (*p* < 0.001; electronic supplementary material, table S19), although the significant effect of elevation was reduced when included in the same model as tree species (*p* = 0.002, *z* = 3.05), and disappeared when included in the same model with ant species (*p* = 0.177, *z* = 1.35). This does not mean that elevation is unimportant; more likely is that elevation drives species composition and ant abundance, which in turn affects herbivory. For *M. subalulata*, herbivory increased with elevation (*p* < 0.001, *z* = 3.61; figure 6*b*,*c*), and this effect disappeared when ant species was included in the model (*p* = 0.076, *z* = 1.78), probably owing to ant species occurrence being co-dependent with elevation. There was no effect of plant occupation by ants on herbivory damage (*p* = 0.103, *z* = 1.63), but when elevation was excluded from the model unoccupied plants showed more herbivory (*p* = 0.027, *z* = 2.21; electronic supplementary material, figure S7).

## 4. Discussion

Few studies have investigated quantitative interaction networks along elevational gradients [38,39], and none, to our knowledge, have studied ant–plant networks in this context. We found that network structure changed with elevation and benefits for plants of ant inhabitation may have been reduced owing to decreased ant recruitment and increased herbivory. Ants of 10 different species inhabited 23 species of terrestrial ant-plants from six families, many of which have not previously been recorded as myrmecophytes (electronic supplementary material, table S1). The high local diversity of ant-hosting plants was not constrained to a particular clade, in common with global patterns [14], and the high ant-plant density indicates significant advantages of ant protection (533 ha^−1^ at 700 m, compared with up to 380 ha^−1^ in central Amazonia [40]). Most plant species hosted multiple species and genera of ants (see also [9]), with numbers of both ant and plant species declining with elevation. As a result, at higher elevations both ants and plants interacted with fewer partners, and networks became more connected, and less specialized. In addition, patterns of plant inhabitation for the most common species suggest reduced partner availability at higher elevations (electronic supplementary material, figure S3*a*–*c*). The most abundant ant-plant, *M. subalulata*, benefitted less at higher elevations, with slower ant recruitment, and higher levels of herbivory. This is, to our knowledge, the first time that context-dependent benefits for domatia-bearing plants have been documented in a whole-community context along an environmental gradient.

Incidence and species richness of both plants and their ant inhabitants declined with elevation, probably owing to lower temperatures and increased precipitation which can limit plant–ant interactions [41]. The upper limit we observed (1600 m) is similar to that for myrmecophytes worldwide [21,42], indicating some fundamental limitation for myrmecophily in plants. Ant communities in general are very strongly limited by elevation, with decreases in ant activity [27,43,44], and in plant-ant colony size [21], presumably owing to thermal limitations [45]. However, the reduction in species richness that we observed was greater than would be expected from reductions in abundance alone (figure 3*a*,*b*), indicating that communities at upper elevations were not just rarefied versions of communities from lower elevations.

The change in ant and plant communities with elevation was accompanied by changes in ant–plant network structure, with the average ant or plant species interacting with fewer partners. However, this apparent increase in partner selectiveness is owing to reduced species richness at higher elevations, as connectance increased, and both network modularity and network specialization (H_2_′) decreased (although the latter result should be treated with caution because specialization only differed from the null expectation at three elevations). This indicates that with fewer partner species to choose from at higher elevations, ants and plants may be less selective in their associations (although note that active partner choice in this system has not been demonstrated). This contrasts with patterns found for seed dispersal and pollinator networks across latitudinal gradients, in which reduced partner availability results in greater specialization [6]. However, decreased specialization with elevation has been observed for leaf miner–parasitoid interaction networks [38]. In contrast with our results, these antagonistic networks showed no change in connectance. The more connected networks that we observed at higher elevations (see [46] for similar results from plant–pollinator networks in the Andes) are probably less sensitive to the loss of species than the less connected networks at lower elevations, because lost species are more likely to be replaced [47], unless core species are lost (e.g. *Anonychomyrma* sp. 13). We found turnover of interactions with elevation, with particular interactions becoming dominant, rather than networks at higher elevations comprising a subset of those from lower elevations (as is the case for cavity nesting hymenoptera and their parasitoids and kleptoparasites [39]). This contrasts with the expected spatial turnover of ant–plant networks, in which the central core of generalist species remains the same [25], supporting the idea that the observed changes are elevation-driven. It is likely that climate plays a key role in these changes, as observed for other mutualistic networks, which are affected by temperature and precipitation [48]. For plants, fewer ant-partner options could result in a suboptimal biotic defence, because with a smaller selection of hosts, it is less likely that a suitable partner will be present. This might in turn reduce plant fitness at certain elevations and ultimately define elevational ranges.

We found some evidence for this reduction in plant protection by ant partners with increasing elevation, accompanied by increased herbivory damage. Although patterns of ant patrolling did not consistently change with elevation, recruitment metrics (first worker recruited, time spent attacking, maximum number of workers observed) indicated a decreased investment in protective behaviour. Similar patterns have been observed for ant predation more broadly at high elevations, with ants becoming less important natural enemies of caterpillars than birds, parasitoid wasps and parasitoid flies [19,22]. Overall, the outcome of ant–plant symbioses is expected to be context-dependent, with our findings indicating that ants provide greater benefits at higher temperatures (within the range that we studied). At lower elevations ants spent similar lengths of time attacking the paper control as they did attacking the surrogate herbivore, but at higher elevations they spent less time on controls and more on surrogate herbivores. This may indicate that only plants at lower elevations receive the benefits of ants removing detritus, vines or other encroaching vegetation (e.g. [49]). The response to the surrogate herbivore also differed between ant species. *Anonychomyrma* sp. 9 was the fastest to detect paper/herbivores, spent longer attacking, and was more abundant on leaves with surrogate herbivores. However, owing to limited overlap in ant elevational ranges, we could not distinguish effects of elevation from effects of species turnover on ant protection of host plants. Yet it is clear that overall, plants were equally well-patrolled, but less well defended at higher elevations. This could partially explain the increase in herbivory damage with elevation, both for the ant-plant community as a whole, and for the species *M. subalulata*.

Such changes in the overall benefits for plants might relate to network structure in two different ways: (i) the smaller number of available ant partners at higher elevations (figure 3*a*) are less likely to include a more beneficial partner (c.f. the ‘sampling effect’ in biodiversity–ecosystem function relationships [50]), or (ii) higher elevation ants in general are less likely to be good partners. Regardless of the driver, these reduced benefits might then cause the breakdown of the mutualism [18], owing to the parallel changes in costs with elevation. Given our findings, it is likely that future anthropogenic-driven changes in the environmental context for these mutualistic networks will alter both interaction network structure, and the balance of costs and benefits for mutualistic partners.

## Supplementary Material

Appendix 1 Figures

## Supplementary Material

Appendix 2 Tables

## Supplementary Material

Appendix 3 Methods

## Data accessibility

Data and R code available from the Dryad Digital Repository (http://dx.doi.org/10.5061/dryad.r9q18) [51].
