# Supplementary material for: Network reorganization and breakdown of an ant–plant protection mutualism with elevation
Source: Proc Biol Sci. 2017 Mar 15;284(1850):20162564. doi: 10.1098/rspb.2016.2564 (PMC5360921; doi:10.1098/rspb.2016.2564)
Supplement: Appendix 1 Figures [file rspb20162564supp1.docx]

**Appendix 1 - Supporting figures**

**
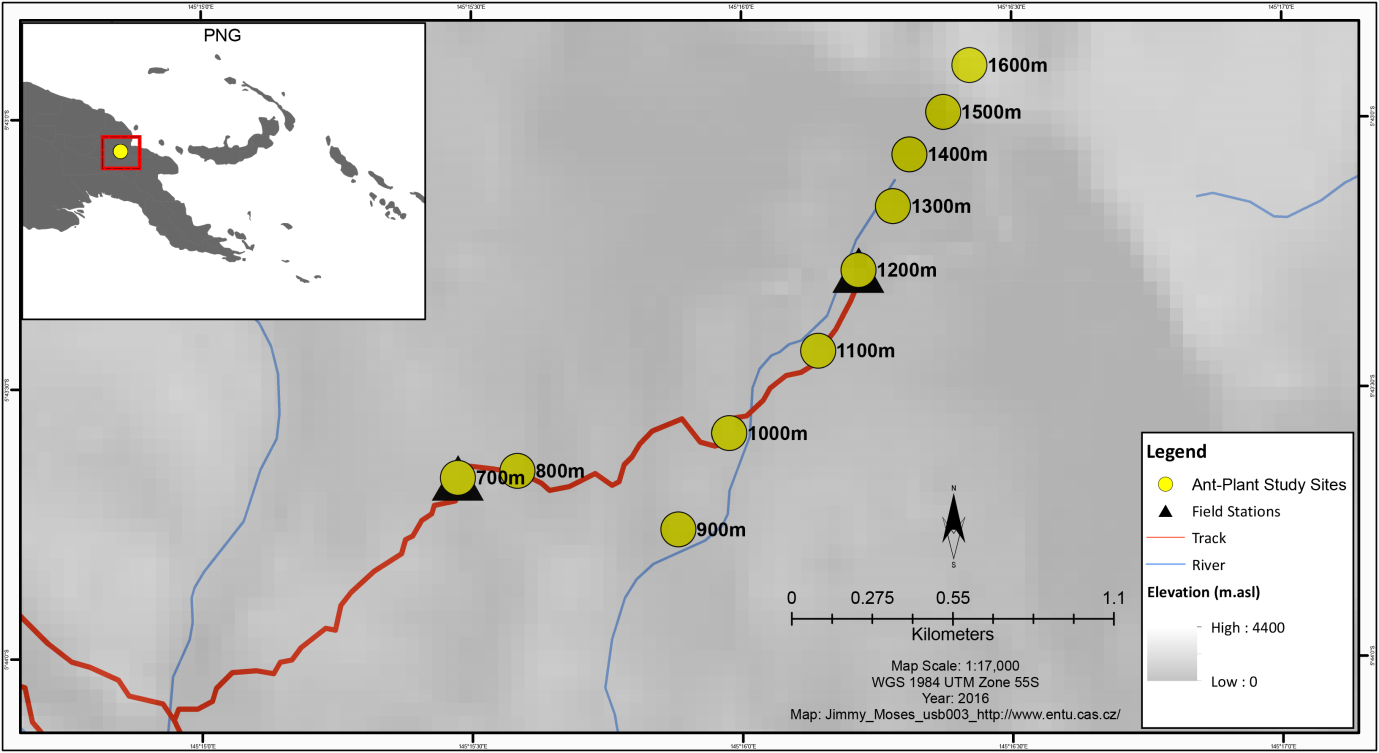
**

**Figure S1.** Schematic map of our study site and sampling points in Madang Province, Papua New Guinea. Yellow circles denote each elevation site in this study, while black triangles are locations of field camps.

Temperature ⁰C

Elevation (m a.s.l)

**Figure S2.** Mean temperatures on Mt. Wilhelm gradient over the course of a year, recorded as part of the IBISCA project “Our Planet Reviewed – Papua New Guinea” (<http://www.laplaneterevisitee.org/en>; data provided by K. Sam). Data loggers (Comet R3120) were placed in standardised vegetation plots at 200, 700, 1200, 1700, 2200, 2700, 3200 and 3700 metres above sea level, and recorded temperature hourly from 30^th^ March 2010 until 1^st^ September 2011.


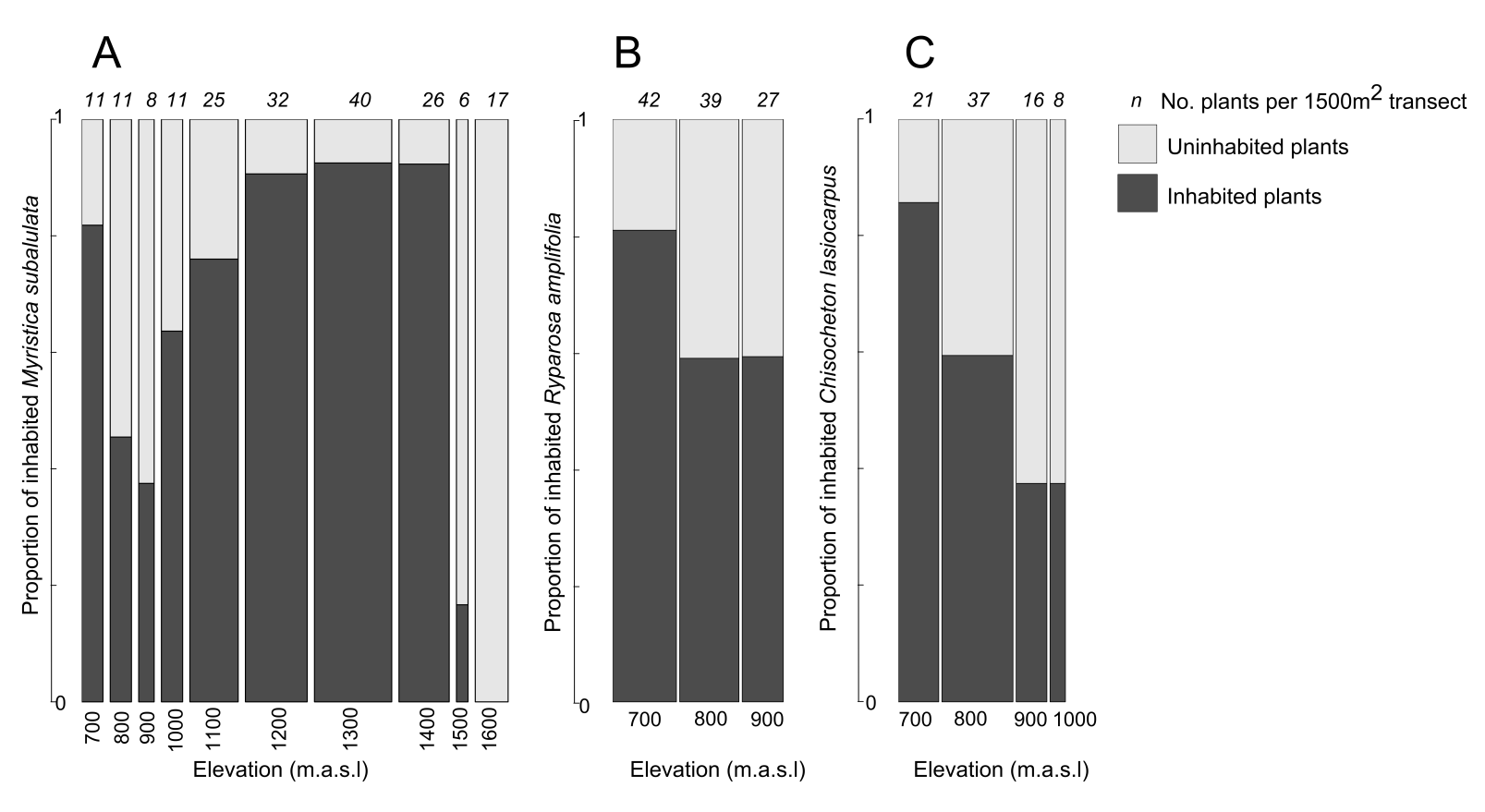


**Figure S3.** Proportion of inhabited and uninhabited plants of the three most common species, A) *Myristica subalulata* (n=187), B) *Ryparosa amplifolia* (n=108) and C) *Chisocheton lasiocarpus* (n=82), across their elevational ranges. Note that bar width scales with *M. subalulata* population size.


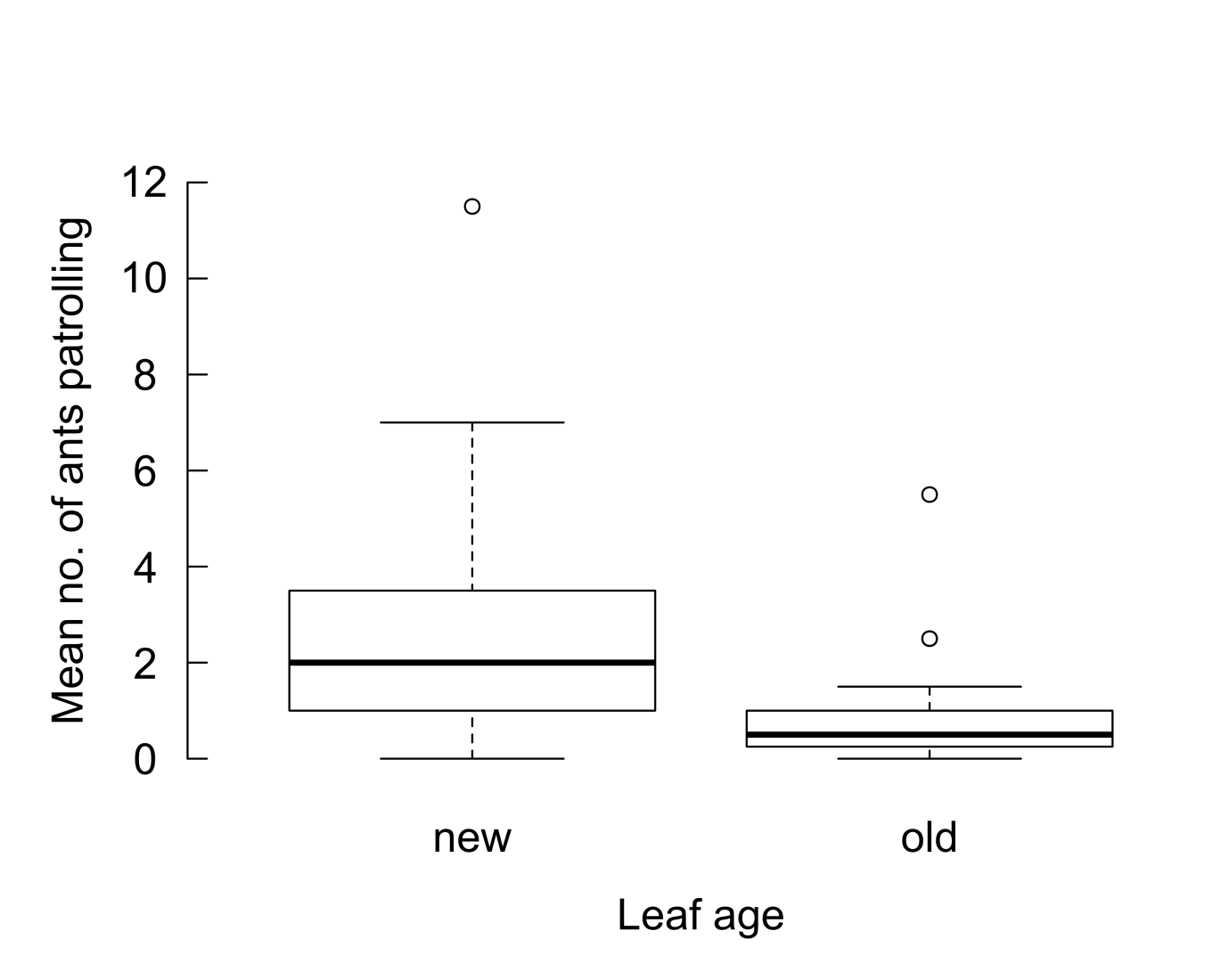


**Figure S4.** Mean number of ants patrolling was greater on new leaves (n=28) than on old leaves (n=78) of *M. subalulata (*repeated measures ANOVA; F=15.1, *P*<0.001, df=24)*.*


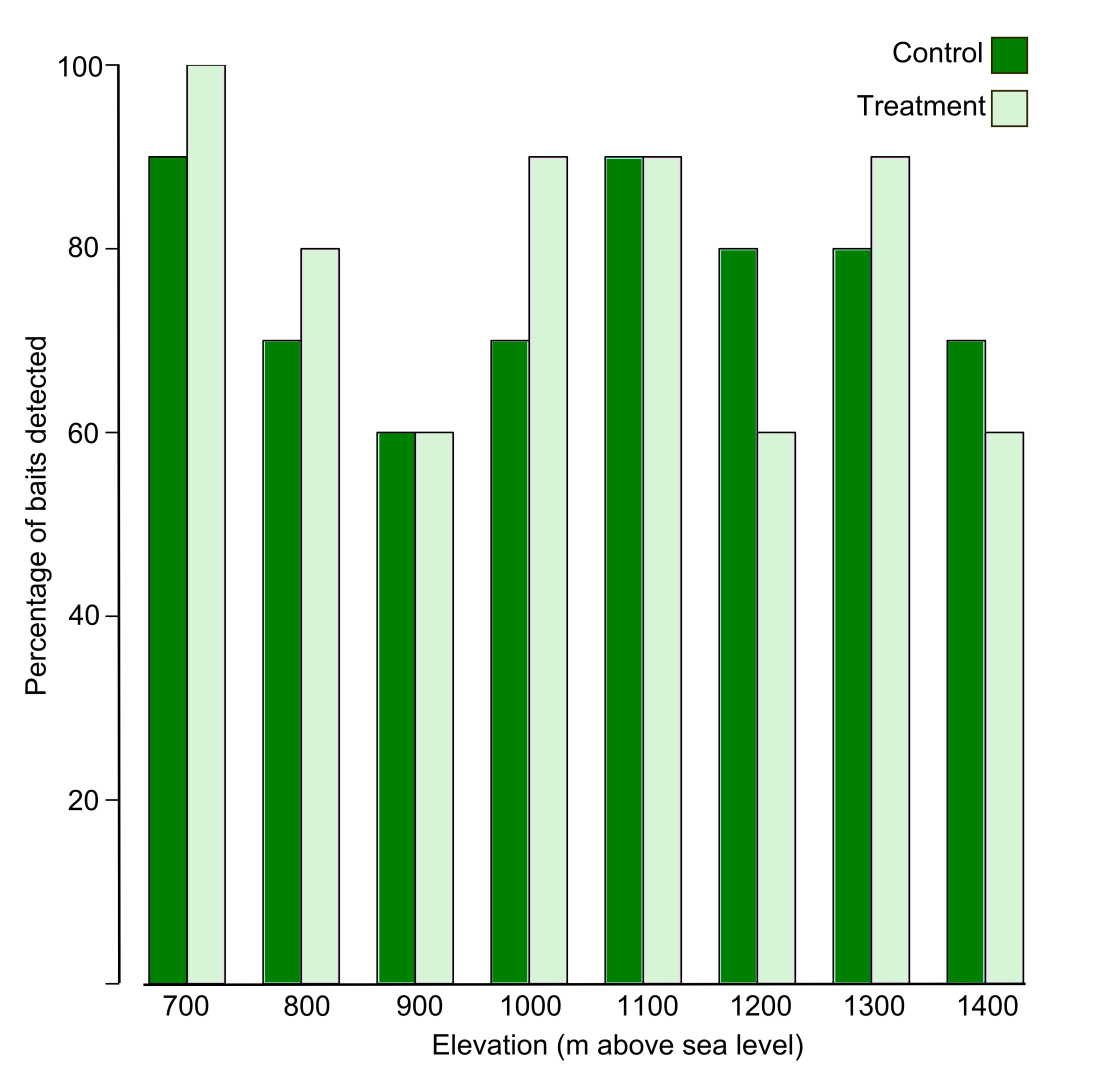


**Figure S5.** Percentage of baits (control=paper square, treatment=worker termite) detected by ants on *Myristica subalulata* leaves from 700 to 1400 metres above sea level (n=10 trees per treatment per elevation).





**Figure S6.** Detection, attack and recruitment of different *Anonychomyrma* ant species to a model herbivore on *Myristica subalulata* host trees. During a ten minute observation period we recorded the following ant responses to a piece of paper (control, light grey) and a live termite (model herbivore, dark grey) pinned to opposing leaves on each plant: A) time taken to first detect paper/model herbivore, B) time spent attacking, C) time taken for the first recruit to contact the paper/model herbivore, D) maximum number of ants observed at one time on experimental leaf. Sample sizes are indicated in italics under each bar (See Baiting ANOVA result tables in Appendix 2).


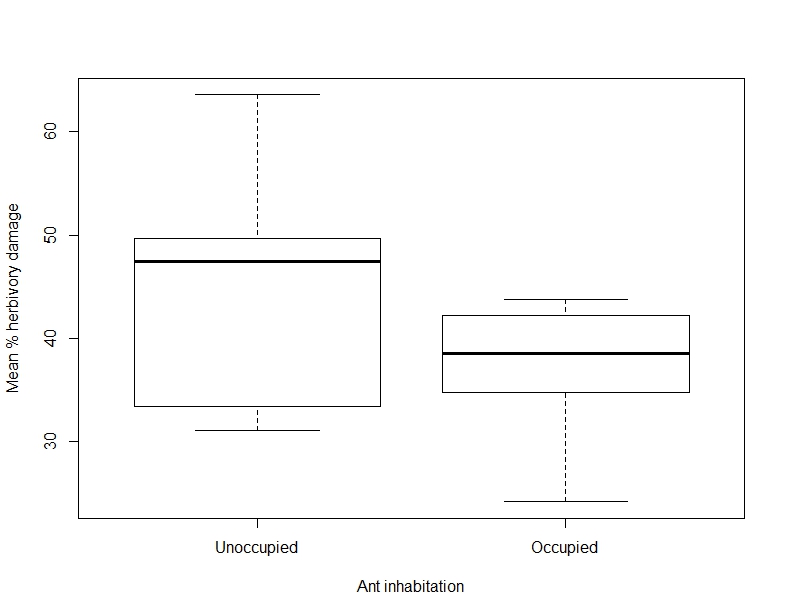


**Figure S7.** Mean percentage herbivory damage of unoccupied (n=89) and occupied plants (n=288) of *Myristica subalulata* (n=187), *Ryparosa amplifolia* (n=108) and *Chisocheton lasiocarpus* (n=82). Unoccupied plants showed more herbivory than those inhabited by ants (ordinal logistic regression; *P*=0.027, z=2.21).
