# Supplementary material for: Network reorganization and breakdown of an ant–plant protection mutualism with elevation
Source: Proc Biol Sci. 2017 Mar 15;284(1850):20162564. doi: 10.1098/rspb.2016.2564 (PMC5360921; doi:10.1098/rspb.2016.2564)
Supplement: Appendix 2 Tables [file rspb20162564supp2.pdf]

Appendix 2 - Supporting Tables

Table S1. The abundance and species identity of all twenty-three species of ant-inhabited plants (n=386) found in our census.

Family	Species	N. ant-inhabited	Previous literature of ant inhabitation
Myristicaceae	*Myristica subalulata* Miq.	134	(Gullan, Buckley & Ward 1993; de Wilde 1998)
Achariaceae	*Ryparosa amplifolia* Mildbr.	73	(Webber *et al.* 2007)
Meliaceae	*Chisocheton lasiocarpus* (Miq.) Valeton	49	
Lauraceae	*Cryptocarya idenburgensis* C.K.Allen	40	
Lauraceae	*Litsea* sp.	18	
Sapindaceae	*Ganophyllum falcatum* Blume	17	
Lauraceae	*Cryptocarya massoy* (Oken) Kosterm.	16	
Lauraceae	*Cryptocarya viridiflora* Kosterm.	8	
Sapindaceae	*Harpullia longipetala* Leenh.	6	
Myristicaceae	*Myristica* sp.	4	
Lauraceae	*Cryptocarya albifrons* Kosterm.	3	
Lauraceae	*Cryptocarya depressa* Warb.	3	
Meliaceae	*Aglaia* sp.	2	
Euphorbiaceae	*Endospermum moluccanum* (Teijsm&Binn.) Kurz.	2	(Letourneau, Arias & Jebb 1993; Letourneau & Barbosa 1999)*
Sapindaceae	*Mischocarpus* sp.	2	
Sapindaceae	*Allophylus cobbe* (L.) Raeusch	2	(Moog *et al.* 2009)**
Meliaceae	*Chisocheton ceramicus* Miq.	1	
Meliaceae	*Chisocheton montanus* P.F. Stevens	1	
Meliaceae	*Dysoxylum excelsum* Blume	1	
Meliaceae	*Dysoxylum parasiticum* (Osbeck) Kosterm.	1	
Myristicaceae	*Horsfieldia* sp.	1	
Euphorbiaceae	*Macaranga* sp.	1	
Euphorbiaceae	*Neoscortechinia forbesii* (Hook.f.) S. Moore	1	

* Published under the synonym *E. labios* **Published under the synonym *Pometia pinnata* (Source: theplantlist.org)

Gullan, P.J., Buckley, R.C. & Ward, P.S. (1993) Ant-tended Scale Insects (Hemiptera: Coccidae: Myzolecanium) within lowland rain forest trees in Papua New Guinea. *Journal of Tropical Ecology*, **9**, 81–91.

Letourneau, D.K., Arias, F. & Jebb, M. (1993) Coping with enemy-filled space: Herbivores on Endospermum in Papua New Guinea. *Biotropica*, **25**, 95–99.

Letourneau, D.K. & Barbosa, P. (1999) Ants, stem borers, and pubescence in Endospermum in Papua New Guinea. *Biotropica*, **31**, 295–302.

Moog, J., Atzinger, K., Hashim, R. & Maschwitz, U. (2009) Do tenants always pay their rent? The Asian ant-plant Pometia pinnata (Sapindaceae) and its leaf domatia provide free access to generalist ants. *Asian Myrmecology*, **2**, 17–32.

Webber, B.L., Moog, J., Curtis, a. S.O. & Woodrow, I.E. (2007) The diversity of ant-plant interactions in the rainforest understorey tree, Ryparosa (Achariaceae): Food bodies, domatia, prostomata, and hemipteran trophobionts. *Botanical Journal of the Linnean Society*, **154**, 353–371.

de Wilde, W.J.J.O. (1998) The myrmecophilous species of Myristica (Myristicaceae) from New Guinea. *Blumea*, **43**, 165–182.

Table S2. The abundance, species identity, and accession number of all ten species of plant-inhabiting ants found in our census. Sequence data is available on BOLD/GenBank, and DNA and ant vouchers are deposited at the Institute of Entomology, Czech Academy of Sciences, České Budějovice. For more details on identification methods see Appendix 3.

Subfamily	Genus	Species	Species Code	No. of trees occupied	Accession no.	Genbank/BOLD BIN code
Dolichoderinae	*Anonychomyrma*	sp. 12	ANON012	99	NA0006	KY095829
Dolichoderinae	*Anonychomyrma*	sp. 13	ANON013	91	NA0008	KY095830
Dolichoderinae	*Anonychomyrma*	sp. 9	ANON009	86	AP700-0250	KY095831
Myrmicinae	*Podomyrma*	sp. 3 aff. *laevifrons* Smith F., 1859	PODO003	47	ASPNA690-09	BOLD:AAG3969
Dolichoderinae	*Anonychomyrma*	*minuta* Donisthorpe, 1943	ANON002	18	ASPNA764-10	BOLD:AAK0675
Dolichoderinae	*Anonychomyrma*	sp. 14	ANON014	8	NA0065	N/A
Formicinae	*Colobopsis*	*quadriceps* Smith, 1859	CAMP013	2	ASPNA686-09	BOLD:AAG4070
Myrmicinae	*Tetramorium*	*bicolor* Viehmyer, 1914	TETR010	2	ASPNA1533-10	BOLD:AAP2923
Formicinae	*Colobopsis*	*vitreus* Smith, 1860	CAMP001	1	ASPNA2352-11	partial sequence
Myrmicinae	*Pheidole*	*amber* Donisthorpe, 1941	PHEI046	1	ASPNA566-09	BOLD:AAG4220

Table S3. Observed and expected specialisation (H2') of networks at each elevation. P values in bold indicate elevations at which observed H2' was significantly different to the expected value. Randomisations were generated using Montecarlo statistics (at http://rxc.sys-bio.net/).

Elevation (m)	H2 observed	H2 randomised (mean)	P	H2 min	H2 max	H2' observed	H2' randomised (mean)
700	2.47897	2.680	**<0.001**	1.825	2.807	0.334	0.129
800	2.46052	2.517	0.134	1.819	2.642	0.220	0.152
900	2.40086	2.496	0.103	1.683	2.704	0.297	0.204
1000	2.12516	2.347	**0.014**	1.779	2.528	0.537	0.241
1100	1.84285	1.982	**0.017**	1.340	2.076	0.317	0.128
1200	2.02171	2.004	0.629	1.307	2.072	0.066	0.088
1300	1.20213	1.166	0.688	0.805	1.202	0.000	0.092
1400	1.05494	1.031	0.599	0.848	1.055	0.000	0.113
1500	NA	NA	NA	NA	NA	NA	NA
1600	NA	NA	NA	NA	NA	NA	NA

Baiting ANOVAs

We provide all model summaries to allow the readers to compare the results of models where covarying factors are included or excluded.

Table S4. Repeated measures ANOVA testing the effect of elevation, tree height and treatment on detection rate.

Coefficient	Df	SS	MS	F	P
Elevation	7	573.6	81.94	1.96	0.078
Treatment	1	4.0	4.00	0.096	0.758
Height	1	2.2	2.20	0.053	0.819
Residuals	53	2214.2	41.78		
Treatment	1	0.3	0.314	0.019	0.89
Residuals	54	884.8	16.385		

Table S5. Repeated measures ANOVA testing the effect of ant species, tree height and treatment on detection rate.

Coefficient	Df	SS	MS	F	P
Species	2	323.3	161.52	3.807	**0.028***
Treatment	1	6.0	6.0	0.142	0.707
Height	1	3.9	3.94	0.093	0.762
Residuals	58	2460.9	42.43		
Treatment	1	0.3	0.314	0.019	0.89
Residuals	54	884.8	16.385		

Table S6. Repeated measures ANOVA testing the effect of elevation, ant species, tree height and treatment on detection rate.

Coefficient	Df	SS	MS	F	P
Elevation	7	573.6	81.94	1.994	0.074
Species	2	120.2	60.12	1.463	0.241
Treatment	1	3.3	3.26	0.079	0.779
Height	1	1.0	0.96	0.023	0.879
Residuals	51	2095.9	41.10		
Treatment	1	0.3	0.314	0.019	0.89
Residuals	54	884.8	16.385		

Table S7. Repeated measures ANOVA testing the effect of elevation, tree height and treatment on attack rate.

Coefficient	Df	SS	MS	F	P
Elevation	7	2104113	300588	4.75	**<0.001*** **
Height	1	29348	29348	0.464	0.498
Residuals	66	4174243	63246		
Treatment	1	373402	373402	18.38	**<0.001*** **
Residuals	74	1503030	20311		

Table S8. Repeated measures ANOVA testing the effect of ant species, tree height and treatment on attack rate.

Coefficient	Df	SS	MS	F	P
Species	2	1493554	74677	11.09	**<0.001*** **
Height	1	32310	32310	0.48	0.491
Residuals	71	4781840	67350		
Treatment	1	373402	373402	18.38	**<0.001*** **
Residuals	74	1503030	20311		

Table S9. Repeated measures ANOVA testing the effect of elevation, ant species, tree height and treatment on attack rate.

Coefficient	Df	SS	MS	F	P
Elevation	7	2104113	300588	4.795	**<0.001*** **
Species	2	184888	92444	1.475	0.237
Height	1	6776	6776	0.108	0.743
Residuals	64	4011927	62686		
Treatment	1	373402	373402	18.38	**<0.001*** **
Residuals	74	1503030	20311		

Table S10. Repeated measures ANOVA testing the effect of elevation, tree height and treatment on maximum abundance.

Coefficient	Df	SS	MS	F	P
Elevation	7	73.74	10.535	3.091	**<0.001*****
Height	1	0.57	0.567	0.166	0.685
Residuals	66	224.94	3.408		
Treatment	1	91.98	91.38	69.03	**<0.001*****
Residuals	74	97.97	1.32		

Table S11. Repeated measures ANOVA testing the effect of ant species, tree height and treatment on maximum abundance.

Coefficient	Df	SS	MS	F	P
Species	2	63.44	31.72	9.598	**<0.001*****
Height	1	1.18	1.18	0.356	0.553
Residuals	71	234.64	3.30		
Treatment	1	91.38	91.38	69.03	**<0.001*****
Residuals	74	97.97	1.32		

Table S12. Repeated measures ANOVA testing the effect of elevation, ant species, tree height and treatment on maximum abundance.

Coefficient	Df	SS	MS	F	P
Elevation	7	73.74	10.535	3.160	**0.006****
Species	2	12.10	6.049	1.814	0.171
Height	1	0.04	0.043	0.013	0.910
Residuals	64	213.37	3.334		
Treatment	1	91.38	91.38	69.03	**<0.001*****
Residuals	74	97.97	1.32		

Table S13. Repeated measures ANOVA testing the effect of elevation, tree height and treatment on time until first recruit.

Coefficient	Df	SS	MS	F	P
Elevation	7	463.9	66.27	2.708	**0.021***
Treatment	1	58.7	58.71	2.399	0.129
Height	1	4.8	4.85	0.198	0.659
Residuals	42	1028.0	24.48		
Treatment	1	21.0	20.96	1.066	0.309
Residuals	36	708.1	19.67		

Table S14. Repeated measures ANOVA testing the effect of ant species, tree height and treatment on time until first recruit.

Coefficient	Df	SS	MS	F	P
Species	2	150.0	74.98	2.659	0.081
Treatment	1	74.9	74.87	2.655	0.110
Height	1	5.2	5.18	0.184	0.670
Residuals	47	1325.4	28.20		
Treatment	1	21.0	20.96	1.066	0.309
Residuals	36	708.1	19.67		

Table S15. Repeated measures ANOVA testing the effect of elevation, ants species, tree height and treatment on time until first recruit.

Coefficient	Df	SS	MS	F	P
Elevation	7	463.9	66.27	2.769	**<0.019***
Treatment	1	58.7	58.71	2.453	0.125
Species	2	75.4	37.70	1.575	0.220
Height	1	0.0	0.04	0.001	0.970
Residuals	40	957.4	23.93		
Treatment	1	21.0	20.96	1.066	0.309
Residuals	36	708.1	19.67		

Table S16. Repeated measures ANOVA testing the effect of elevation, tree height and treatment on the detection time of *Anonychomyrma* sp.9 to check for within species effects of elevation.

Coefficient	Df	SS	MS	F	P
Elevation	4	349.4	87.34	2.110	0.138
Treatment	1	6.8	6.77	0.164	0.692
Height	1	19.8	19.84	0.479	0.501
Residuals	13	538.1	41.40		
Treatment	1	2.12	2.115	0.129	0.724
Residuals	18	295.51	16.417		

Table S17. Repeated measures ANOVA testing the effect of elevation, tree height and treatment on the detection time of *Anonychomyrma* sp.12 to check for within species effects of elevation.

Coefficient	Df	SS	MS	F	P
Elevation	5	153.8	30.76	1.174	0.360
Treatment	1	31.2	31.16	1.190	0.290
Height	1	49.9	49.92	1.906	0.184
Residuals	18	471.4	26.19		
Treatment	1	6.1	6.119	0.353	0.558
Residuals	22	381.0	17.317		

Ordinal Logistic Regression results

We provide all model summaries to allow the readers to compare the results of models where covarying factors are included or excluded.

Table S18. Results of ordinal linear regression model (*clmm* function, *ordinal* package) testing the effect of elevation and tree height on herbivory damage of all ant-inhabited trees ≤ 5 m in height (n=7584 leaves; 507 trees).

Coefficients	Estimate	Std Error	Z value	P
Elevation	0.14	0.003	54.6	**<0.001** ***
Height	0.13	0.003	37.7	**<0.001** ***

Table S19. Results of ordinal linear regression model (*clmm* function, *ordinal* package) testing the effect of ant species and tree height on herbivory damage of all ant-inhabited trees ≤ 5 m in height (n=7584 leaves; 507 trees).

Coefficients	Estimate	Std Error	Z value	P
ANON009	-0.98	0.40	-2.44	**0.015***
ANON012	-1.13	0.40	-2.87	**0.004****
ANON013	-0.17	0.39	-0.44	0.658
PODO003	-0.86	0.42	-2.04	**0.042***
Height	0.17	0.07	2.39	**0.017***

Table S20. Results of ordinal linear regression model (*clmm* function, *ordinal* package) testing the effect of tree species on herbivory damage of all ant-inhabited trees ≤ 5 m in height (n=7584 leaves; 507 trees).

Coefficients	Estimate	Std Error	Z value	P
Cryptocarya idenburgensis	-0.76	0.27	-2.81	**0.005** **
Cryptocarya massoy	-0.43	0.40	-1.06	0.289
Ganophyllum falcatum	-1.68	0.31	-5.42	**<0.001*****
Litsea sp.	-0.09	0.34	-0.27	0.789
Myristica subalulata	0.87	0.22	3.97	**<0.001** ***
Ryparosa amplifolia	-0.60	0.24	-2.47	**0.013** *

Table S21. Results of ordinal linear regression model (*clmm* function, *ordinal* package) testing the effect of elevation, ant species and tree species on herbivory damage of all ant-inhabited trees ≤ 5 m in height (n=7584 leaves; 507 trees).

Coefficients	Estimate	Std Error	Z value	P
Elevation	0.00	0.00	0.77	0.441
ANON009	-0.83	0.37	-2.27	**0.023** *
ANON012	-0.97	0.36	-2.66	**0.008** **
ANON013	-0.18	0.36	-0.49	0.621
PODO003	-0.37	0.41	-0.89	0.372
Cryptocarya idenburgensis	-0.89	0.33	-2.70	**0.007** **
Cryptocarya massoy	-0.55	0.46	-1.18	0.237
Ganophyllum falcatum	-2.01	0.42	-4.74	**<0.001** ***
Litsea sp.	-0.09	0.38	-0.24	0.812
Myristica subalulata	0.28	0.31	0.90	0.368
Ryparosa amplifolia	-0.64	0.26	-2.46	**0.014** *

Table S22. Results of ordinal linear regression model (*clmm* function, *ordinal* package) testing the effect of elevation and tree species on herbivory damage of all ant-inhabited trees ≤ 5 m in height (n=7584 leaves; 507 trees).

Coefficients	Estimate	Std Error	Z value	P
Elevation	0.13	0.04	3.05	**0.002** **
Cryptocarya idenburgensis	-1.13	0.29	-3.87	**<0.001** ***
Cryptocarya massoy	-0.83	0.42	-2.00	**0.045** *
Ganophyllum falcatum	-2.39	0.38	-6.23	**<0.001** ***
Litsea sp.	-0.33	0.35	-0.94	0.346
Myristica subalulata	0.32	0.28	1.13	0.360
Ryparosa amplifolia	-0.59	0.24	-2.47	**0.013** *

Table S23. Results of ordinal linear regression model (*clmm* function, *ordinal* package) testing the effect of elevation, tree height and ant species on herbivory damage of all ant-inhabited trees ≤ 5 m in height (n=7584 leaves; 507 trees).

Coefficients	Estimate	Std Error	Z value	P
Elevation	0.07	0.05	1.35	0.177
Height	0.16	0.07	2.29	**0.022** *
ANON009	-0.86	0.41	-2.09	**0.036** *
ANON012	-0.99	0.41	-2.45	**0.014** *
ANON013	-0.31	0.40	-0.78	0.437
PODO003	-0.59	0.46	-1.29	0.198

Table S24. Results of ordinal linear regression model (*clmm* function, *ordinal* package) testing the effect of elevation, tree height and ant species on herbivory damage of *Myristica subalulata* in our baiting experiment.

	Estimate	Std. Error	Z value	P
Elevation	0.11	0.06	1.78	0.076
Height	-0.02	0.08	-0.27	0.786
ANON012	-0.17	0.23	-0.77	0.441
ANON013	0.24	0.36	0.67	0.504

Table S25. Results of ordinal linear regression model (*clmm* function, *ordinal* package) testing the effect of elevation and tree height on herbivory damage of *Myristica subalulata* in our baiting experiment.

	Estimate	Std. Error	Z value	P
Elevation	0.15	0.04	3.61	**<0.001 *****
Height	-0.04	0.08	-0.53	0.598

Table S26. Results of ordinal linear regression model (*clmm* function, *ordinal* package) testing the effect of ant species and tree height on herbivory damage of *Myristica subalulata* in our baiting experiment.

	Estimate	Std. Error	Z value	P
ANON012	-0.03	0.22	-0.15	0.877
ANON013	0.73	0.25	2.96	**0.003****
Height	-0.07	0.08	-0.85	0.397
