# Supplementary material for: Network reorganization and breakdown of an ant–plant protection mutualism with elevation
Source: Proc Biol Sci. 2017 Mar 15;284(1850):20162564. doi: 10.1098/rspb.2016.2564 (PMC5360921; doi:10.1098/rspb.2016.2564)
Supplement: Appendix 3 Methods [file rspb20162564supp3.pdf]

Appendix 3 - Supplementary Methods & Ant Photos

Ant identification and barcoding

Ants were stored in 99% ethanol and examined under Olympus SZX7 microscope at magnification 12-84x. Since taxonomic knowledge of ants from the region is poor at the species level, ants were first sorted to genera and morphospecies. We compared them with reference collections from Klimes et al. (2015) and from the international expedition *Our Planet Reviewed* at Mt. Wilhelm (Leponce et al, 2017), which sampled ants in the same or nearby sites. All morphospecies were also compared with the ant image databases New Guinea Ants and Antweb (www.newguineants.org, www.antweb.org). Some species within the *Anonychomyrma* genus, which is in need of taxonomic revision, were only possible to identify to morphospecies level. For each (morpho)species, a representative worker was sequenced for the mitochondrial gene cytochrome c oxidase I (COI), either previously as part of the Barcode of the life project (sequence deposited in BOLD database, http://www.barcodinglife.org) or newly for the purpose of this study (deposited in Genbank, See Table S2). The BOLD sequences were obtained at the Biodiversity Institute of Ontario, University of Guelph using a standard protocol described in Smith et al. (2005), with respective primers used listed for each sample in the BOLD database. The rest of the samples were obtained at the Institute of Entomology, where total genomic DNA was extracted using the GenomicDNAKit Tissue (Geneaid Biotech Ltd., New Taipei City, Taiwan) following the manufacturer's protocol. COI was then amplified using published primers and polymerase chain reaction conditions (Folmer et al, 1994). DNA sequences were assembled, edited and aligned in Geneious version 6.1. The *Anonychomyrma* sequences were also compared with sequences from previously collected specimens of the genus from New Guinea and Australia (M. Janda, P. Matos-Maraví unpublished data; and available data in Genbank and BOLD). DNA and ant vouchers are deposited at the Institute of Entomology, Biology Centre of Czech Academy of Sciences, České Budějovice.

References

Folmer et al (1994). DNA primers for amplification of mitochondrial cytochrome c oxidase subunit I from diverse metazoan invertebrates. Molecular Marine Biology and Biotechnology 3(5), 294-299.

Klimes P., Fibich, P., Idigel C. & Rimandai M. (2015) Disentangling diversity of arboreal ant communities in tropical forest trees. PLoS ONE 10(2): e0117853.

Leponce M. *et al.* (2017). Land module of Our Planet Reviewed - Papua New Guinea: aims, methods and first taxonomical results. In: Insects of Mount Wilhelm, Papua New Guinea. Ed. by T. Robillard F. Legendre C. Villemant & M. Leponce. Mémoires du Muséum national d'Histoire naturelle, Paris, pp 595 (book chapter).

Smith, M. A., Fisher, B. L., and Hebert, P. D. (2005). DNA barcoding for effective biodiversity assessment of a hyperdiverse arthropod group: the ants of Madagascar. Philosophical Transactions of the Royal Society of London. Series B, Biological Sciences 360(1462), 1825–1834.

Frontal and lateral view of ant species

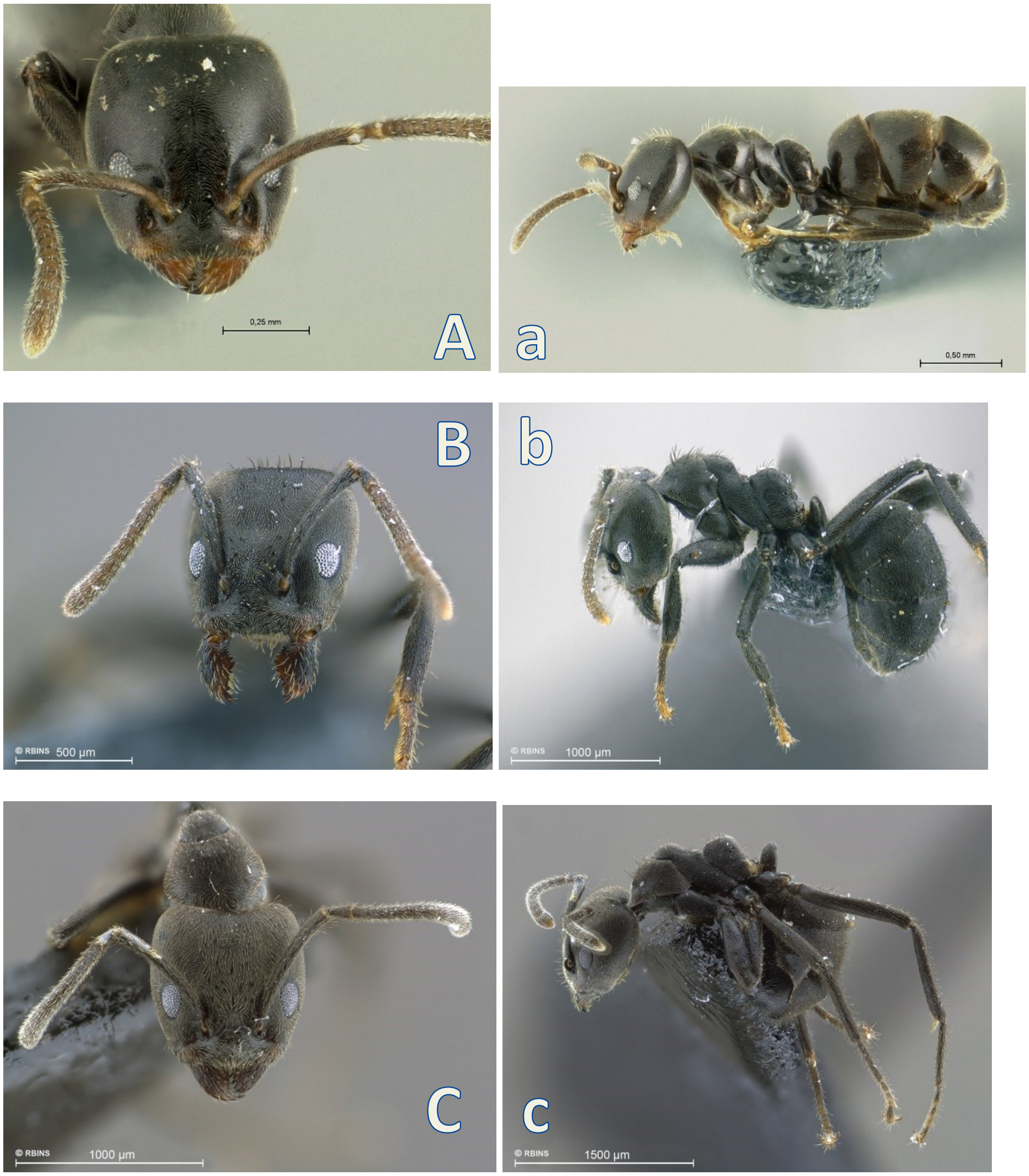

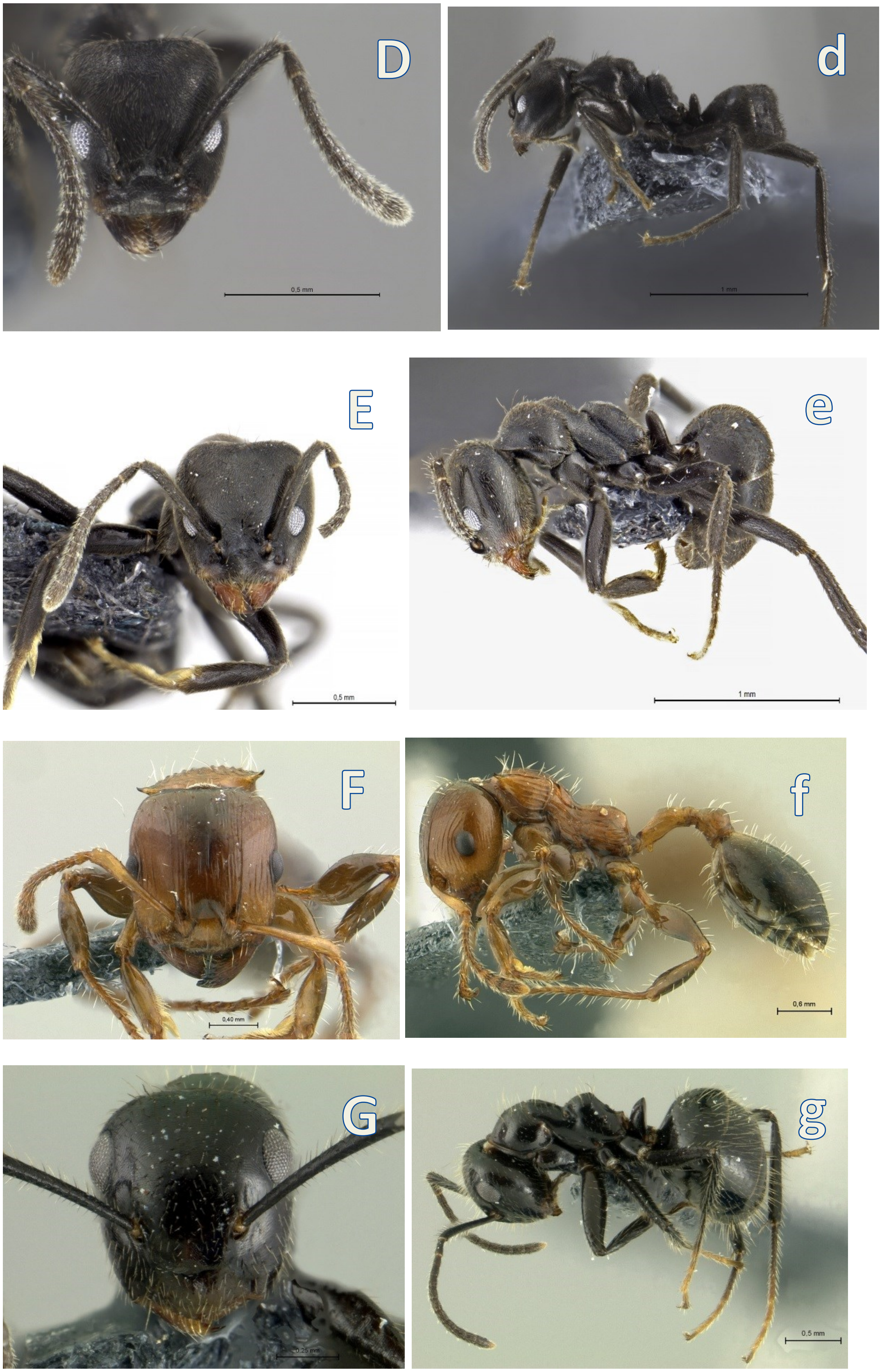
D
0,5 mm
d
1 mm
E
0,5 mm
e
1 mm
F
0,40 mm
f
0,6 mm
G
g
0,5 mm

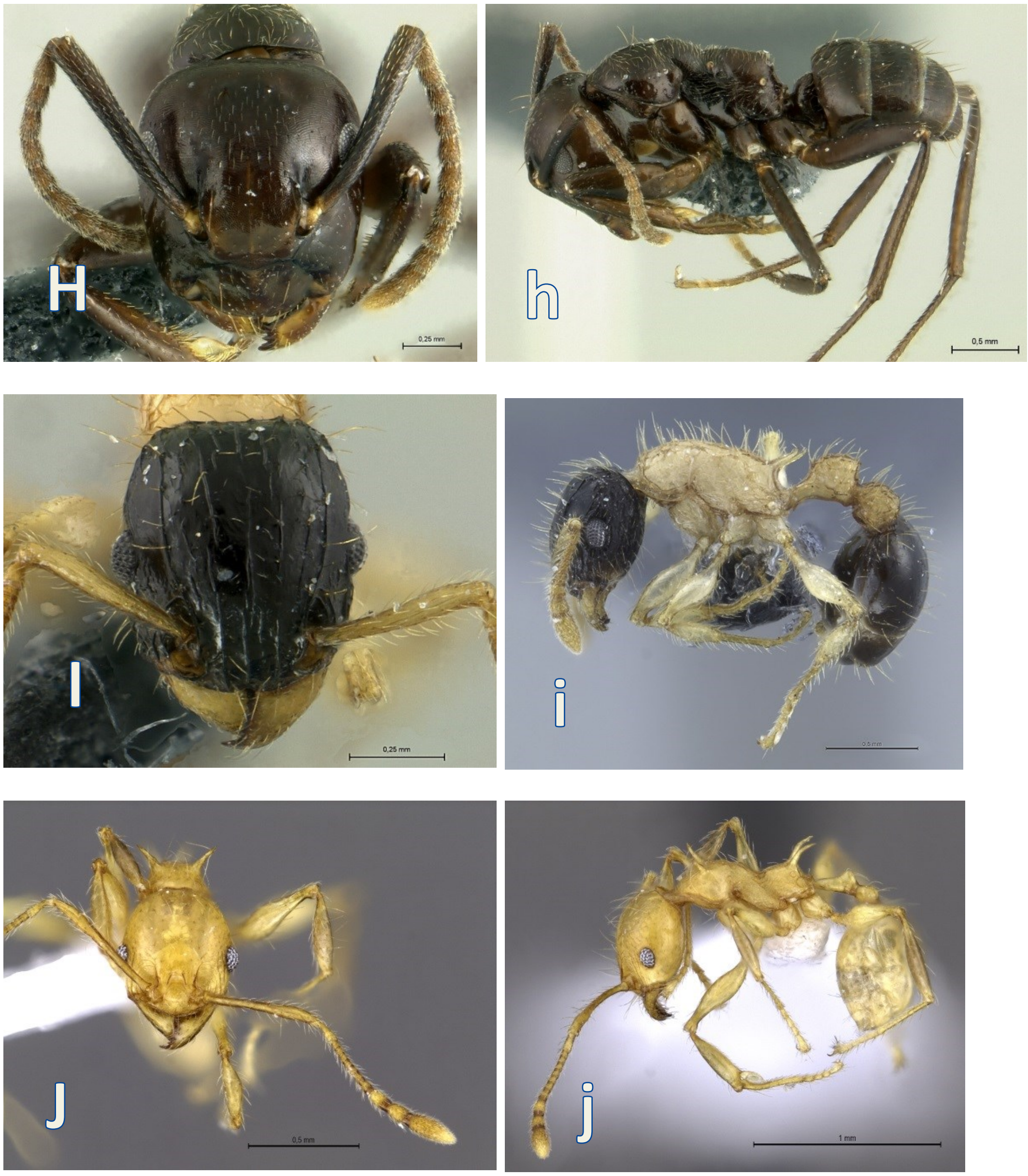

Photos Aa-Jj: Head (uppercase letter) and lateral (lowercase) views of *Anonychomyrma* sp.2 (Aa), *Anonychomyrma* sp. 9 (Bb), *Anonychomyrma* sp.12 (Cc), *Anonychomyrma* sp. 13 (Dd), *Anonychomyrma* sp.14 (Ee), *Podomyrma* sp.3 (Ff), *Colobopsis vitrea (Gg)*, *Colobopsis quadriceps (*Hh), *Tetramorium bicolor* (Ii), *Pheidole amber* (Jj). See table S2 for further details. Photos courtesy of the Institute of Entomology, Biology Centre of the Czech Academy of Sciences (A, D-J)) and the Royal Belgian Institute of Natural Sciences (B-C). Ant specimens are deposited at the Institute of Entomology, Biology Centre CAS, České Budějovice.
